# Structure of the Current Sheet in the 11 July 2017 Electron Diffusion Region Event

**DOI:** 10.1029/2018JA026028

**Published:** 2019-02-27

**Authors:** Rumi Nakamura, Kevin J. Genestreti, Takuma Nakamura, Wolfgang Baumjohann, Ali Varsani, Tsugunobu Nagai, Naoki Bessho, James L. Burch, Richard E. Denton, Jonathan P. Eastwood, Robert E. Ergun, Daniel J. Gershman, Barbara L. Giles, Hiroshi Hasegawa, Michael Hesse, Per‐Arne Lindqvist, Christopher T. Russell, Julia E. Stawarz, Robert J. Strangeway, Roy B. Torbert

**Affiliations:** ^1^ Space Research Institute Austrian Academy of Sciences Graz Austria; ^2^ Space Science Center University New Hampshire Durham NH USA; ^3^ Mullard Space Science Laboratory University College London Dorking UK; ^4^ Institute of Space and Astronautical Science Japan Aerospace Exploration Agency Sagamihara Japan; ^5^ Goddard Space Flight Center NASA Greenbelt MD USA; ^6^ Southwest Research Institute San Antonio TX USA; ^7^ Department of Physics and Astronomy Dartmouth College Hanover NH USA; ^8^ The Blackett Laboratory Imperial College London London UK; ^9^ Department of Astrophysical and Planetary Sciences University of Colorado Boulder Boulder CO USA; ^10^ Department of Physics and Technology University of Bergen Bergen Norway; ^11^ Royal Institute of Technology Stockholm Sweden; ^12^ Department of Earth, Planetary, and Space Sciences University of California, Los Angeles Los Angeles CA USA

**Keywords:** magnetic reconnection, electron diffusion region, current sheet, Magnetospheric Multiscale (MMS)

## Abstract

The structure of the current sheet along the Magnetospheric Multiscale (MMS) orbit is examined during the 11 July 2017 Electron Diffusion Region (EDR) event. The location of MMS relative to the X‐line is deduced and used to obtain the spatial changes in the electron parameters. The electron velocity gradient values are used to estimate the reconnection electric field sustained by nongyrotropic pressure. It is shown that the observations are consistent with theoretical expectations for an inner EDR in 2‐D reconnection. That is, the magnetic field gradient scale, where the electric field due to electron nongyrotropic pressure dominates, is comparable to the gyroscale of the thermal electrons at the edge of the inner EDR. Our approximation of the MMS observations using a steady state, quasi‐2‐D, tailward retreating X‐line was valid only for about 1.4 s. This suggests that the inner EDR is localized; that is, electron outflow jet braking takes place within an ion inertia scale from the X‐line. The existence of multiple events or current sheet processes outside the EDR may play an important role in the geometry of reconnection in the near‐Earth magnetotail.

## Key Points

- Current sheet structure in electron diffusion region is deduced using multipoint measurements from MMS to infer reconnection parameters
- Rate of electron acceleration along the out‐of‐plane electric field is consistent with theory of meandering electrons in 2‐D reconnection
- Consistency with 2‐D reconnection theory is found in reconnection electric field from electron pressure gradient and spatial extent of EDR

## 1 Introduction

Magnetic reconnection is a fundamental plasma process where magnetic energy is converted to plasma kinetic and thermal energy by changing the topology of the magnetic field. Reconnection takes place in thin current sheets where electrons decouple from the magnetic field, that is, the electron diffusion region (EDR), which are embedded in the larger ion diffusion region (IDR) where ions are also decoupled from the magnetic field. Electron dynamics in the EDR have been extensively studied using particle‐in‐cell (PIC) simulations (e.g., Bessho et al., 2014; Fu et al., 2006; Hesse et al., 2001; 2011; Hoshino et al., 2001; Huang et al., 2010; Nakamura et al., 2016; Ng et al., 2011, 2012; Pritchett, 2001, Shay et al., 2001, 2007; Shuster et al., 2015). These studies determined how the electrons moving into the diffusion region exhibit meandering motion, are accelerated by the out‐of‐plane electric field, and are eventually magnetized in the outflow region. Analytical formulae have been developed relating the changes in the EDR electron distribution function or moment characteristics to the reconnection electric field and scales of the EDR (Hesse et al., 2001; 2011, Bessho et al., 2014, Nakamura et al., 2016).

Observations of 3‐D electron distribution functions with sufficient temporal resolution to study the EDR became available with the launch of the Magnetospheric Multiscale (MMS) mission (Burch, Moore, et al., 2016). The first EDR measurements of asymmetric reconnection were reported by Burch, Torbert, et al. (2016), followed by numerous EDR observations in the magnetopause and magnetosheath. As for the magnetotail, where symmetric reconnection is expected, Torbert et al. (2018) reported for the first time EDR signatures of a near‐antiparallel reconnection event observed on 11 July 2017, between 22:30 and 22:40 UT. Multiple‐crescent distributions were found and the aspect ratio of the diffusion region was determined to be 0.1–0.2, consistent with many simulations of fast reconnection. It was concluded that the effects of turbulence and associated fluctuations on the electron dynamics are small in the observed EDR. Nakamura et al. (2018) performed a fully kinetic simulation of the same event. They showed remarkable consistency with the MMS EDR observations. The normalized and unnormalized reconnection rates from the simulation (Nakamura et al., 2018) were consistent with the observed values of *E*_M_, 0.15–0.2 and 2–3 mV/m, respectively, obtained by Genestreti et al. (2018), who used out‐of‐plane (*M*) directions based on different methods to determine the current sheet orientation for this event. All these studies concluded that MMS encountered a magnetic reconnection EDR in a near‐2‐D current sheet configuration around 22:34:03 UT.

In this paper, we examine the spatial structure of the current sheet and the evolution of the electron distribution function for the EDR event on 11 July 2017 at 22:34:03 UT based on multipoint analysis of MMS magnetic, electric field, and electron data. We compare the deduced reconnection parameters with the predicted values from theoretical models of 2‐D reconnection. The estimated electric field due to nongyrotropic term of the pressure tensor and the rate of the acceleration of meandering electrons are shown to be consistent with theoretical estimations. The smaller scales of the observed inner EDR than expected from 2‐D PIC simulations suggest that existence of multiple events or current sheet processes outside EDR may play important role in the geometry of reconnection in the near‐Earth magnetotail.

## 2 Current Sheet Crossing

**Figure 1:**
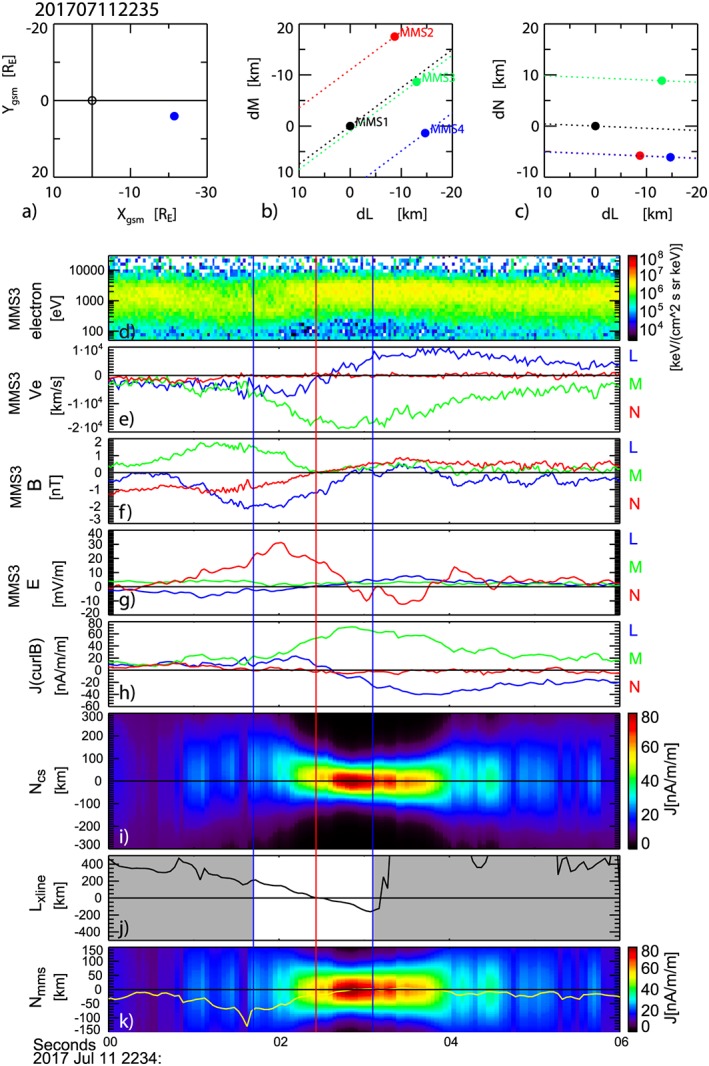
Magnetospheric Multiscale (MMS) spacecraft location and overview of the thin current sheet observation near the X‐line. (a) Location of MMS in GSM *X‐Y* plane and location of the four MMS spacecraft relative to MMS1 in (b) *L‐M* plane and (c) *L‐N* plane. (d) Electron energy spectra, (e) electron flow, (f) magnetic field, (g) electric field from MMS 3 spacecraft, (h) current density obtained from the curlometer method, (i) current density distribution relative to MMS location deduced from the current sheet model, (j) location of the X‐line relative to the location of MMS from linear gradient method, and (k) current density distribution and location of the spacecraft within the model current sheet deduced by the 1‐D linear gradient method by also taking into account the gradient along *L* (yellow trace). The *L*, *M*, and *N* components in (e)–(h) are plotted in blue, green, and red. The vertical red line indicates the X‐line crossings for MMS 3, that is, 22:34:02.4 UT. The blue vertical lines denote the time interval when X‐line monotonically moves tailward crossing MMS. Outside this region is shown as gray area in (j).

On 11 July 2017, MMS crossed the magnetotail current sheet region around 22:34 UT. A weak substorm with multiple intensifications of 200 nT in the westward electrojet commenced at 22:33 UT. MMS was located at (*X, Y, Z*)_GSM_ = (−21.6, 4.1, 3.8) *R*_E_ and the interspacecraft distances were within ~18 km (Figures 1a–1c). This spacecraft separation is comparable to the electron inertial scale outside the current sheet and near the EDR center (1 *d*_e_ ~ 31 km, for this event). The overview of the entire reconnection event has been described in detail by Nakamura et al. (2018), Genestreti et al. (2018), and by Torbert et al. (2018). Overview plot of MMS3 observation is also shown in Figure S1 in the supporting information. MMS was located in the Southern Hemisphere when the tailward fast ion flow started at around 22:32 UT. The crossing of the current sheet with EDR signatures took place around 22:34 UT (interval between the two vertical bars) when the reversal of *V*_X_ of both ions, electrons, and *B*_Z_ took place as shown in Figure S1g–S1i. The orbit of the MMS relative to the X‐line for this event was shown in Torbert et al. (2018); Figure 1). Upstream ion and electron beta inferred from the average values during the short interval outside the reconnection jet: 22:33:23–22:33:28 UT were 0.5 ± 0.09 and 0.1 ± 0.02, respectively. These values are similar to those before the start of the tailward flow (Figure S1f). After several north‐south crossings, MMS was located in the Northern Hemisphere when the fast ion flows subside around 22:38 UT (Figures S1g and S1h). During the flow reversal intervals, strong north‐south electric field, *E*_Z_, directed toward the center of the current sheet, are visible (Figure S1j), as expected for a Hall electric field in a thin current sheet. MMS3 observations during the thin current sheet crossing interval between 22:34:00 and 22:34:06 are shown in Figures 1d–1g: electron energy spectra and velocity data from the fast plasma instrument (Pollock et al., 2016) with time resolution of 30 ms, magnetic field data with 128 sample/s from the fluxgate magnetometer (Russell et al., 2014), and electric field data from the double‐probe instrument (Ergun et al., 2016; Lindqvist et al., 2016) with 32 sample/s. MMS3 crossed the vicinity of the X‐line (the reversal in the normal component of the magnetic field to the current sheet) closest to the neutral sheet among the four spacecraft. We use in this study current sheet *LMN* coordinates, where *L* = (0.9482, −0.255, −0.1893), *M* = (0.1818, 0.9245, −0.3350), and *N* = (0.2604, 0.2832, 0.9230) in the GSE coordinate system. This coordinate system was determined based on a hybrid method. That is, *L*, which corresponds to the reconnection field direction tangential to the current sheet, is obtained from the direction of maximum variance of the electron velocity using MMS 3 data between 22:34:02 and 22:34:04 UT. *M* is the cross product of the normal component of the current sheet determined using the maximum directional derivative of B (MDD‐B) technique of Shi et al. (2005) and *L*. *N* is the cross product of *L* and *M*. This coordinate system combines the results from the independent data sets, that is, magnetic field maximum gradient and electron velocity maximum variance, which were most accurately determined (Genestreti et al., 2018). This coordinate turned out to be almost identical to the maximum variance coordinate determined from the electron velocity (Genestreti et al., 2018). The *M* component of the electric field in this coordinate provides most consistent values expected from the simulated reconnection electric field by Nakamura et al. (2018). (More detail on the differences among the coordinate systems determined from different techniques has been given by Genestreti et al. (2018)).

Figures 1d and 1e show that the reversal of *B*_N_ from southward to northward occurs at around 22:34:02.4 UT (indicated by the red vertical line) when a reversal in *Ve*_L_ from tailward to earthward is also taking place, which are signatures of an X‐line moving tailward. The accompanying reversal in *E*_L_ shown in Figure 1g is expected due to the change in MMS3 location from tailward to earthward of the X‐line. The crossing of the neutral sheet (reversals in *B*_L_) between 22:34:02.8 and 22:34:03.8 UT has nearly simultaneous reversals in *E*_N_ that agree with the expected profile of the Hall electric field, which should be directed toward the neutral sheet. These signatures show that the X‐line moved tailward across the spacecraft, while MMS approached the current sheet center from the southern hemisphere. *B*_M_ was mainly enhanced in the southern hemisphere (*B*_L_ < 0) preceding the X‐line outside EDR, which is consistent with quadrupole magnetic field. Yet when MMS approached the current sheet center within the electron jet region, *B*_M_ was rather small as is expected in the center of EDR.

The enhanced *V*_eM_ < 0 profile nearly coincides with the strong enhancement in *M* component of the current density (Figure 1h) obtained by the linear gradient (curlometer) technique (Chanteur, 1998) using the magnetic field data from the four MMS spacecraft. This indicates development of the electron current sheet. To examine the evolution of the current sheet, we determined the current sheet thickness, *D*, and location of the current sheet center, *N*_*0*_ *,* during this interval using a current sheet model:

(1) $$B_{L\_\text{model}\_t}\left(N\left(t\right)\right)=B_{0t}\tanh\left(\frac{N\left(t\right)-N_{0t}}{D_{t}}\right)\;$$

Here *B*_*0t*_ is the magnetic field outside the current sheet. By assuming that the magnetic field pressure dominates outside the current sheet and the pressure balance across the current sheet holds at each time of the four‐point measurements, *B*_*0t*_ is determined from the observed total pressure, which is the sum of ion, electron, and magnetic pressure, and vacuum permeability, *p*_*0*_ and ***μ***_*0*_, as *B*_*0t*_ = (2 ***μ***_*0*_ *p*_*0*_)^1/2^. The subscript *t* denotes the model parameters for time “*t*”. For each *t*, we obtained the thickness, *D*_*t*_, and current sheet center, *N*_*0t*_, of a modeled current sheet using data at the barycenter of the four spacecraft, *N*_bc_ and *B*_*L_*bc_, and the estimated linear gradient, *∂B*_*L*_ */∂N*. Instead of directly using *∂B*_*L*_ */∂N*, we created a virtual data point at *B*_*L_*bc_ + *∂B*_*L*_ */∂N* × ∆*N* and *N*_bc_ + ∆*N* so that *D*_*t*_ and *N*_*0t*_ can be easily determined from simple manipulations of (1). For ∆*N* we used the average spatial scale of the four‐point measurements, that is, four‐spacecraft average of the *N* component of the inverse of the reciprocal vector (Chanteur, 1998). In order to exclude high‐frequency disturbances, we used 16 sample/s (“survey”) data with an adjusted spin‐axis offset, which were used also for the determination of the coordinate system by Genestreti et al. (2018). Figure 1i shows the evolution of the *N* dependence of the current density relative to the MMS 3 location at the time of the neutral sheet crossing, that is, 22:34:02.4 UT, based on the current sheet model applied to the curlometer data as described above. As implied by the magnetic field and current density variations (Figures 1f and 1h), an intense electron‐scale (several tens of kilometer thick) current sheet is present near the X‐line with the thinnest current sheet observed after the crossing of the X‐line in the *L* direction. The thin current is a very transient feature (~1 s). Both, before the X‐line crossing, around 22:34:01.5 UT and after the X‐line crossing, after 22:34:04.5 UT, MMS stayed near the equator, *B*_*L*_ = 0. Yet a thin intense current was not visible during these time periods. Hence, the thin observed current sheet containing the EDR was either temporal or spatially localized in *L*. We note that for an X‐line picture one would expect the thinnest current sheet to be observed at the X‐line. Here the delay is most likely related to the fact that at the beginning of the X‐line crossing, MMS was too far outside (south) from the current sheet center to deduce the correct profile of the thin electron scale current sheet.

Since the tailward moving X‐line and the vertical motion of the current sheet are most likely independent processes, we deduce the spacecraft motion of the X‐line in the *L* direction and relative to the current sheet in the *N* direction, separately. The motion of the X‐line is determined based on an assumption that the X‐line is a stationary structure and that *B*_*N*_ is constant with respect to *N*. The latter assumption follows for an approximately one‐dimensional structure, for which the *N* direction is the magnetic field minimum variance direction. Indeed similar profile of *B*_*N*_ reversal among the spacecraft (shown later) supports the assumption of a static structure. Then the motion in the *L* direction can be obtained from the observed temporal change in *B*_*N*_ and the gradient of *B*_*N*_ along *L* determined from the curlometer method. Its location at *t*, *L*_xline_ (*t*), can then be determined by integrating the deduced velocity as

(2) $$L_{\text{xline}}\left(t\right)=\int_{t_{\mathrm{ref}}}^{t}\left(\frac{\partial B_{N}\left(t^{\prime }\right)}{\partial t^{\prime }}\right){\left(\frac{\partial B_{N}\left(t^{\prime }\right)}{\partial L}\right)}^{-1}dt^{\prime }+L_{\text{xline}}\left(t_{\mathrm{ref}}\right)\;$$

This method has been successfully used to obtain the current sheet density profile during its rapid vertical crossings (Nakamura et al., 2006; Runov et al., 2006) and is a simplified 1‐D version of a more comprehensive method developed by Shi et al. (2006), where the coordinate system is also simultaneously determined. The estimated *L* coordinate of the X‐line is shown in Figure 1j relative to MMS3 spacecraft using the X‐line crossing time of MMS3 as the reference time, *t*_ref_. That is, *L*_xline_ (*t*_ref_) = 0. Here we used the location and magnetic field values at the barycenter of the spacecraft. Continuous tailward motion of the X‐line, expected from the *B*_*N*_ reversal, can be seen for only a 1.4‐s interval around the X‐line crossing time. The blue vertical lines at 22:34:01.7 UT and 22:34:03.1 UT in Figure 1 indicate the beginning and the end of this interval. Due to the earthward motion of magnetic structures preceding and afterward, our simple assumption of a static X‐line moving tailward fails and finding a reasonable estimate for the motion of the X‐line becomes difficult outside this time interval (shown as gray area in Figure 1j).

In a similar way, the *N* coordinate of the MMS spacecraft relative to the current sheet is determined as

(3) $$N_{\mathrm{mms}\left(i\right)}\left(t\right)=\int_{t_{\mathrm{ref}}}^{t}\left[\left(\frac{\partial B_{L}\left(t^{\prime }\right)}{\partial t^{\prime }}\right)-\left(\frac{\partial B_{L}\left(t^{\prime }\right)}{\partial L}\right)\left(\frac{\partial L}{\partial t^{\prime }}\right)\right]\;\;{\left(\frac{\partial B_{L}\left(t^{\prime }\right)}{\partial N}\right)}^{-1}dt^{\prime }+N_{\mathrm{mms}\left(i\right)}\left(t_{\mathrm{ref}}\right)\;$$

Note that unlike for the estimation of the speed of the X‐line motion, where we assumed that the *B*_*N*_ is constant across the current sheet, we take into account the change in the current sheet profile as can be seen in Figure 1h; that is, *B*_*L*_ changes both along *L* and *N*. This effect is added in the second term inside the bracket and can be expressed using the modeled location as

(4) $$\left(\frac{\partial B_{L}\left(t^{\prime }\right)}{\partial L}\right)\left(\frac{\partial L}{\partial t^{\prime }}\right)\;{\left(\frac{\partial B_{L}\left(t^{\prime }\right)}{\partial N}\right)}^{-1}=\frac{N_{\text{model}\_t\prime +\mathrm{∆t}}\left(B_{L}(t^{\prime }\right))-N_{\text{model}\_t\prime }(\left(B_{L}\left(t^{\prime }\right)\right)}{\mathrm{∆t}}$$

The obtained *N* coordinate of the MMS barycenter relative to the current center using (3) is shown in Figure 1k in yellow curve. It should be noted that when only the first term in the bracket of (3) is used, the predicted spacecraft location will be oddly placed at off equator in the northern hemisphere for the times when *B*_L_ = 0, around 22:34:00.5 UT. Hence, we use the MMS orbit calculated using the *L* dependence deduced from the current sheet models applied to each time as given in (3).

## 3 Change in the Electron Distribution Function

**Figure 2:**
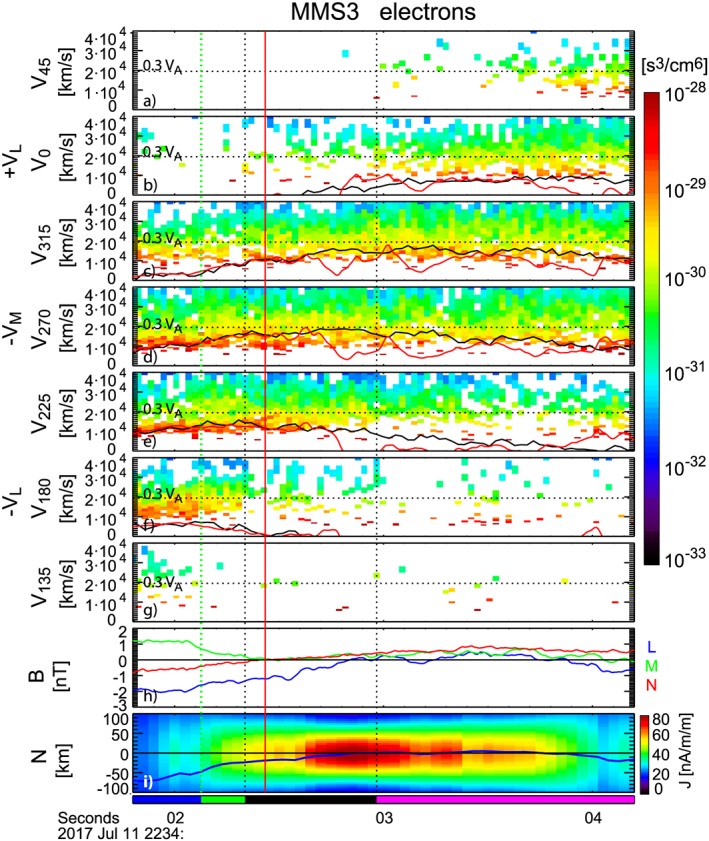
Magnetospheric Multiscale 3 (MMS3) electron velocity spectra near the X‐line crossing and the location of the spacecraft relative to the current sheet. Velocity spectra cuts in directions within the *L‐M* plane along the (a) *V*_45_, (b) *V*_0_, or +*V*_L_, (c) *V*_315_, (d) *V*_270_, or −*V*_M_, (e) *V*_225_, (f) *V*_180_, or –*V*_L_, and (g) *V*_135_ directions shown in Figure 3a and explained in the text. (h) *L* (blue), *M* (green), and *N* (red) components of the magnetic field. (i) *N* coordinate of MMS3 within the model current sheet. In (a)–(g) the bulk velocity and the E × B drift components along the direction of the cuts are shown as black and red curves, and the horizontal dotted line indicates the velocity level of 0.3 *V*_A_ = 19,500 km/s. The vertical red line indicates the crossing of the X‐line in the *L* direction by MMS 3 at 22:34:02.4 UT. The vertical dotted lines show the demarcation of the four regions with different characteristics of the velocity distribution function marked with bars at the bottom, representing the region outside the thin current sheet (blue), the tailward outflow jet region (green), the inner Electron Diffusion Region (black), and the earthward outflow jet region (magenta).

**Figure 3:**
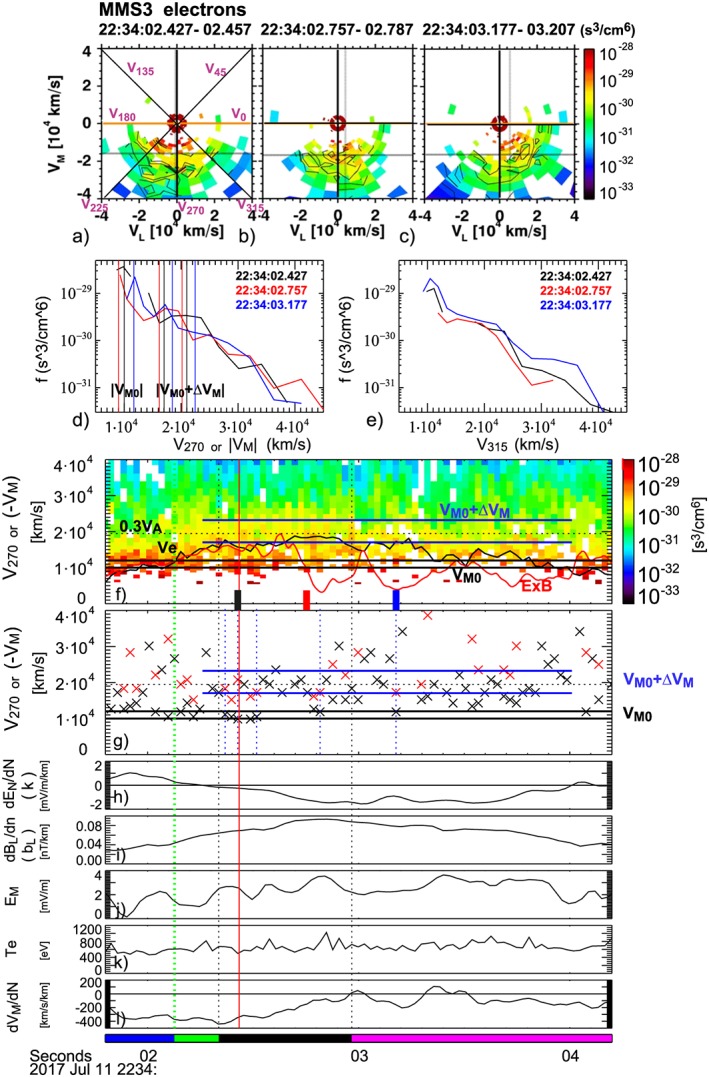
Magnetospheric Multiscale 3 (MMS3) electron velocity distribution function and gradient parameters. Two‐dimensional cuts in the velocity distribution function in the *L‐M* plane for (a) 22:34:02.427–22:34:02.457 UT (showing *V*_*α*_ directions for the velocity distribution cuts displayed in Figure 2), (b) 22:34:02.757–22:34:02.787 UT, (c) 22:34:03.177–22:34:03.207 UT, and 1‐D cut along (d) the *V*_270_ (i.e., −*V*_M_) direction and (e) the *V*_315_ direction for the distribution functions shown in (a)–(c). (f) Velocity spectra cuts along the *V*_270_ or −*V*_M_ direction, (g) velocity of the first (black cross) and second (red cross) peak in the spectra cuts along the *V*_270_ direction, (h) d*E*_N_/d*N*, (i) d*B*_L_/d*N*, (j) *E*_M_, and (k) electron temperature from MMS 3, and (l) d*V*_M_/d*N*. The gradient parameters in (h), (i), and (l), are obtained from the linear gradient method (Chanteur, 1998). The vertical lines in (d) and horizontal lines in (f) and (g) indicated as *V*_M0_ are used to estimate the predicted ranges of the second peak in the distribution function, *V*_M0_ + *∆V*_M_, shown as vertical (d) and horizontal (f, g) lines using model from Bessho et al. (2014) for meandering electrons accelerated by the reconnection electric field. The thin blue dotted lines in (g) indicate those times when both first and second peaks in the distribution function are identified in the data as predicted by the model (see more detailed explanation in the text). The electron bulk velocity (black) and the E × B drift (red) are also shown in (f). The other vertical lines in (f)–(l) and the bottom bars are the same as in Figure 2.

Multiple crescent and triangular shapes in electron distribution functions, identified near the X‐line crossing from the distribution function in field‐aligned coordinates, were reported for this event by Torbert et al. (2018). These features are characteristic of the nongyrotropic electron distribution in the diffusion region. These triangular shapes and discrete striation in the distribution functions in the EDR have been shown to rotate toward the outflow direction in the PIC simulations (Bessho et al., 2014; Bourdin, 2017; Le et al., 2016; Shuster et al., 2015). To reconstruct the spatial distribution of these specific distribution function patterns within the EDR thin current sheet, we examined the temporal evolution of cuts in the electron distribution function in the *L‐M* plane as shown in Figure 2 for MMS 3. Figures 2a–2g shows different portions of the distribution. The angle, *α*, for the cuts, in the *V*_α_ direction is the angle in the *L‐M* plane, where 0° is along +*L* direction, as given also in Figure 3a. The width of these angle cuts are ±15° around the *L‐M* plane and ±15° for each direction within the *L‐M* plane. The bulk velocity and ***E*** *×* ***B*** drift components along the direction of the cuts are shown as black and red curves. The dotted lines indicate the velocity level of 0.3 *V*_A_ = 19,500 km/s, where *V*_A_ is the electron Alfven velocity using the density values of *n* = 0.03 cm^−3^ and *B* = 12 nT as a reference value outside the current sheet before the reconnection event.

The plots of cuts in the velocity spectra, Figures (2a–2g), show clear changes in their properties. Near the X‐line, the dawnward (−*M*) velocity reaches up to about 0.3 *V*_A_, as can be seen in the *V*_270_ spectra plot (Figure 2d). This maximum dawnward velocity is consistent with the results from the PIC simulation, performed with mass ratio of 400, for this event using input parameters close to the observed values (Nakamura et al., 2018). The phase space density around 0.3 *V*_A_ is largest for the *V*_270_ (−*V*_M_) cut, but is enhanced over a wider azimuthal range between 22:34:02.34 UT and 22:34:02.96 UT, delineated by two vertical black dotted lines marking the ends of the black bar at the bottom of Figure 2. Characteristic 2‐D distribution function profiles during this interval are shown in Figures 3a and 3b. Preceding this interval, enhanced phase space density up to around 0.3 *V*_A_ can be seen in the *V*_180_ spectra plot (Figure 2f), indicating a tailward outflow jet is evolving. This outflow region is marked by a green bar at the bottom of the figure and starts from 22:34:02.13 UT (green vertical line), indicating entrance into the hotter plasma region from a lower‐energy region as is expected for a separatrix crossing. The transition time coincides with the encounter of the stronger current sheet (Figures 2h and 2i). The region outside the thin current sheet is marked with blue bar at the bottom of the figure. Enhanced earthward outflow up to around 0.3 *V*_A_ can be seen in the *V*_0_ spectra plot (Figure 2b) after 22:34:02.96 UT, marked by a magenta bar at the bottom of the plot. An example of the 2‐D distribution function cut during this time is shown in Figure 3c. Similar changes in the distribution function were observed by the other spacecraft (see supporting information Figures S2–S4).

The effects from the triangular shape or multiple crescent distribution discussed by Torbert et al. (2018) can be most clearly seen in the *V*_270_ spectra plot near the X‐line where distinct peaks in the phase space density below and above the bulk flow component can be seen (Figure 2d or 3f). Example 2‐D cuts in the *L‐M* plane of such distribution are shown in Figures 3a–3c for selected times indicated by the thick black, red, and blue tics, respectively, in Figure 3f. Figure 3d shows 1‐D cuts of the distributions shown in Figures 3a–3c along *V*_270_, that is, the *−V*_M_, direction, where multiple peaks can be identified. Based on studies of the electron distribution near the EDR using PIC simulations, it has been found that the electrons undergoing meandering bounces in the EDR will create such multiple striations (multiple crescents) in the distribution function in the *L‐M* plane (Bessho et al., 2014; Bourdin, 2017; Ng et al., 2011, 2012; Shuster et al., 2015). The rotation of the multiple crescent distribution toward the outflow direction, as can be seen in Figures 3a–3c, is also consistent with the simulation (Bourdin, 2017; Shuster et al., 2015). An analytic formula was obtained relating the velocity gain, ∆*V*_M_, due to acceleration by the electric field, *E*_M_, while bouncing within the current sheet due to finite *B*_L_ and drifting dawnward due to the Hall electric field *E*_N_. Assuming linear variation of *B*_L_ and *E*_N_ in the current sheet, that is, *E*_N_ ***= −**k N* and *B*_L_ ***=*** *b*_L_ *N*, this velocity gain (or the distance between the first and the second stripes in the distribution function) is expressed for the center of the current sheet as:

(5) $$∆V_{M}=\left\{{\left[1+\frac{3\pi }{2}{\left(\frac{e{E_{M}}^{2}}{b_{L}m_{e}}\right)}^{1/2}{\left|V_{M0}+\frac{k}{b_{L}}\right|}^{-3/2}\right]}^{2/3}-1\right\}\left|V_{M0}+\frac{k}{b_{L}}\right|,$$

where *e* is the elementary charge and *m*_*e*_ is the electron mass (Bessho et al., 2014).

Using the observed gradient parameters, *k* and *b*_*L*_ (Figures 3h and 3i), which are obtained from the linear gradient method (Chanteur, 1998), and *E*_M_ (Figure 3j) we calculated ∆*V*_M_ from the above formula (Bessho et al., 2014). Since the peaks in the distribution functions are created by the incoming electrons, then undertaking meandering motions across the current sheet, we used average values of *E*_M_ *, k*, and *b*_L_ near the center of the current sheet instead of local values. Here we defined the center of the current sheet by the interval with negative *k* values. Outside this region, the amplitude of the Hall‐electric field is decreasing, k > 0, meaning *E*_N_ is smaller outside the current sheet (Figure 3h). Furthermore, the dawnward electron velocity gradient (Figure 3l) profile suggests that the electron currents started to decay more gradually outward as expected in the outer edge of the current sheet.

The lowest velocity peaks in the distribution function detected each time are plotted as black crosses in Figure 3g. Due to sparse plasma, the low energy peaks, which are visible in the blue and black curves in Figure 3d, could not be detected for many of the cuts during this time interval, such as the case for the red curve in Figure 3d. Over the time interval of the EDR crossing, however, there are low energy peaks recurrently visible between 10,000 and 12,000 km/s, which are shown as two horizontal lines in Figure 3f and 3g indicated as *V*_M0_. This corresponds approximately to the value of *V*_eM,_ (= *(**E** × **B**)*_M_)***,*** near the outer edge of the current sheet around 22:34:02.2 UT. The two horizontal blue lines in Figure 3f and 3g show the estimated range of *V*_M0_ *+* ∆*V*_M_ for *V*_M0_ = −10,000 to −12,000 km/s and taking into account the standard deviation of 2,200 km/s of ∆*V*_M_ estimation using the average field parameters. For those times when the lowest velocity peak was less than 17,000 km/s, which is the lower value of *V*_M0_ *+* ∆*V*_M_, the second peak values are also plotted with red crosses in Figure 3g. It can be seen that either a black or a red cross exists most of the times inside the estimated range of *V*_M0_ *+* ∆*V*_M_, in particular, in the inner EDR. There are five instances when both *V*_M0_ *+* ∆*V*_M_ and *V*_M0_ are detected within the expected range and indicated by vertical dotted lines in Figure 3g. The black and the blue curves in Figure 3d are from such instances when an observed *V*_M0_ value can be used to estimate the *V*_M0_ *+* ∆*V*_M_ range. For the red curve, *V*_M0_ = −9,000 km/s, which was the velocity of the maximum phase space density, is used to estimate the range of *V*_M0_ *+* ∆*V*_M._ As can be seen in Figures 3d, 3f, and 3g, there are quite a number of times that enhancement in the phase space density near a velocity of *V*_M0_ *+* ∆*V*_M_ is visible, most clearly in the inner EDR but also some in the outflow region. In the outflow region, the VDF is rotated toward *V*_L_ so that the 1‐D cut along *V*_315_ direction (Figure 3e) shows more clearly multiple components, which are associated with the multicrescent signatures (blue trace). Interestingly, during the time when MMS3 is close to the equator, where the local *(**E** × **B**)*_M_ due to the Hall field is small, *V*_M0_ *+* ∆*V*_M_ matches the bulk velocity, *V*_eM_ quite well_._ The consistency of these observed peaks with the value of *V*_M0_ *+* ∆*V*_M_ from (5) obtained by Bessho et al. (2014) indicates that we can quantitatively identify electron meandering motion in the EDR thin current sheet. Such stripes can be seen in the inner EDR region (black bars at the bottom of Figures 2 and 3) as well as in the Earthward outflow jet region (magenta bars). The results support the conclusion by Bessho et al. (2018) that reconnection electric field can be estimated from the second peak in the velocity distribution functions such as shown in Figure 3d.

## 4 Spatial Structure of Inner EDR and Reconnection Electric Field

**Figure 4:**
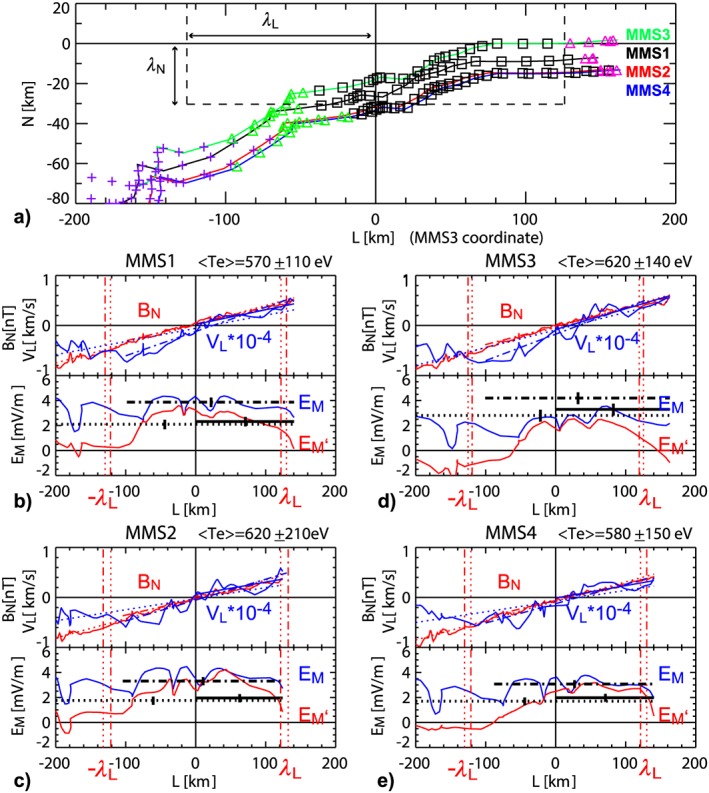
Orbit of the four spacecraft relative to X‐line and changes in reconnection parameter along *L*. (a) Location of the four spacecraft between 22:34:01.7 and 22:34:03.1 UT relative to the X‐line observed by Magnetospheric Multiscale 3 (MMS3). The colored symbols show the four different velocity distribution function patterns (purple: outside separatrix, green: tailward outflow jet region, black: inner EDR, magenta: earthward outflow jet region). The color of the curves represents the different spacecraft (black: MMS1, red: MMS2, green: MMS3, blue: MMS4). (b) *B*_N_,(red) and *V*_eL_, (blue; upper panel) and *E*_M_,(blue) and *E*_M_’ (red; lower panel) along *L* measured relative to the X‐line for MMS1. Linear fits to the curves are shown for *B*_N_ and *V*_eL_ (upper panel) to obtain the gradients, ∂*V*_L_/∂L and ∂*B*_N_/∂l for estimating λ_L_ and *E*_M,NG_, respectively. The linear fit and corresponding parameter estimations are performed for the interval 22:34:01.7–22:34:03.1 UT (dotted lines), for data |*N*| < 50 km (dash‐dotted lines) and for *L >* 0 km (solid lines). Average temperature and its standard deviation for the interval 22:34:01.7–22:34:03.1 UT are given above the panels. The errors of the *E*_N,NG_ estimation calculated using this temperature fluctuations and the standard error of regression slope are given as vertical ticks on the horizontal bars in the lower panels. (c)–(e) are same as (b) but for MMS2, MMS3, and MMS4, respectively.

We further examine the spatial structure of the current sheet near the X‐line using data between 22:34:01.7 UT and 22:34:03.1 UT when continuous tailward motion of the X‐line was detected and information about the location of the spacecraft relative to the X‐line and the current sheet can be deduced (Figure 1). Figure 4a shows the four spacecraft orbit around the X‐line. The colored symbols along the orbit represents the types of 30‐ms electron distribution function using the same color scheme as was used for the horizontal bars in Figures 2 and 3. The transition from tailward outflow region (green bar) to the inner EDR (black bar) takes place at 25–35 km south of the equator, suggesting that the entry into the EDR on the tailward side of the X‐line is due to the relative northward motion of the spacecraft toward the current sheet center. The half thickness of the inner EDR was about one electron inertia length. On the other hand, the transition from the inner EDR to Earthward outflow (outer EDR) region (magenta) takes place near the equator for all spacecraft, at a distance of 120–160 km away from the X‐line. So this distance is the dimension along the outflow direction. The average aspect ratio of this region is then ~0.2, which is consistent with the values obtained by Torbert et al. (2018) based on timing analysis and the electron current and velocity profiles of MMS3.

Hesse et al. (1999) showed that the scale size of the electron (inner) diffusion, where the electric field is dominated by the nongyrotropic electron pressure, is determined by the trapping length of electrons in field reversals and can be determined from *λ*_N_ = [(2*m*_e_ *T*_e_/(*e* ^2^(∂*B*_L_/∂*N*)^2^)]^1/4^ for the scale perpendicular to the current sheet and *λ*_L_ = [(2*m*_e_ *T*_e_/(*e* ^2^(∂*B*_N_/∂*L*)^2^)]^1/4^ for the scale along the electron outflow direction. Using the average value of Te between 22:34:01.7 UT and 22:34:03.1 UT for all spacecraft <*T*_e_ > = 600 ± 150 eV, and <∂*B*_N_/∂*L* > = 0.0036 ± 0.0008 nT/km, <∂*B*_L_/∂N > = 0.062 ± 0.022 nT/km, we obtain *λ*_N,ave_ = 30 ± 7 km and *λ*_L,ave_ = 125 ± 23 km. These values are comparable to the expected thickness and length of the inner EDR from the observed location of the different types of the electron distribution function as shown in Figure 4a.

Assuming that (a) *B*_N_ increases linearly outward from the X‐line *B*_N_ *= (*∂*B*_N_/∂*L*) *L;* (b) electrons become magnetized at the edge of the diffusion region, *λ*_L_; (c) the inflow and outflow electron density is approximately constant; and using the simplified formula for the nongyrotropic electron pressor tensor of Kuznetsova et al. (1998), Hesse et al. (1999) found that the electric field due to the nongyrotropic pressure can be expressed as follows:

(6) $$E_{M,\mathrm{NG}}=-\frac{1}{n_{e}e}\left(\frac{\partial P_{\mathrm{eLM}}}{\partial L}+\frac{\partial P_{\mathrm{eNM}}}{\mathrm{\partial N}}\right)\approx \frac{1}{e}\frac{\partial V_{\mathrm{eL}}}{\partial L}\;\sqrt{2m_{e}T_{e}}\;$$

where *P*_eLM_ and *P*_eNM_ are the off‐diagonal components of the pressure tensor. The validity of this formula has been shown in different PIC simulations (Dorfman et al., 2008; Hesse et al., 1999; Lu et al., 2013; Nakamura et al., 2016).

Using the location of the spacecraft, we compare values found using this formula with the observed electric field for each spacecraft separately. The upper panels in Figures 4b–4d show the profile of the observed *B*_N_ (red) and *V*_eL_ (blue). Here *L* = 0 is the location of the X‐line encountered by each spacecraft. Linear fits for *B*_N_ are shown as dotted lines, for which all the data between 22:34:01.7 UT and 22:34:03.1 UT are used. The dash‐dotted lines show the results for which only data within |*N*| < 50 km are used to limit the data points to those near the center of the current sheet. Using *(*∂*B*_N_/∂*L*) deduced from the slope for each spacecraft, we obtained *λ*_L_ and drew the distance, *λ*_L_, on either side of the X‐line as vertical lines in Figures 4b–4d. The *λ*_L_ values are between 119 and 132 km and are therefore comparable to *λ*_L,ave._ In the same way, we calculate linear fits to *V*_eL._ Here we also determined the gradient of *V*_eL_ for *L* > 0 (Earthward of the X‐line) and plotted the fit as a solid line in addition to finding fits for the same intervals as were used for *B*_N_. Using the obtained *(*∂*V*_L_/∂L), we estimate *E*_M,NG_ and show the resulting values with horizontal bars (dash dotted including *L* < 0, and solid for just *L* > 0) having a length corresponding to the data interval used in the lower panels in Figure 4b–4d. These panels also show the measured electric field, *E*_M_ (blue) and the electric field in the electron frame, *E*_M_′ = (***E*** + ***V***_e_ × ***B***)_M_ (red). The temperature used for the calculation is shown above the upper panels. The calculated errors for *E*_M,NG_ using the standard deviation of the temperature and the standard error of the regression slope are shown as vertical ticks on the bars.

Near the X‐line in the inner diffusion region, *E*_M_ is close to *E*_M_′ and the electric field is expected to be due to the nongyrotropic part of the pressure tensor. For the tailward side (*l* < 0), this region extends only to about *L* = −60 to −100 km where *E*_M_′ drops out associated with the spacecraft location 1–1.5 *λ*_N,ave_ south of the current sheet. The drop in *E*_M_′ at the Earthward side, on the other hand, coincides with the *L* distance becoming ~*λ*_L._ The estimated *E*_M,NG_ for the three different intervals agree within the error bars for MMS3, while *E*_M,NG_ estimated using condition |*N*| < 50 km is slightly larger than the other two estimates for MMS1, MMS2, and MMS4. Nonetheless, it can be seen that most of these estimates provide comparable values to the observed *E*_M,_ or *E*_M_′ in the inner EDR. Hence, the spatial scale of the inner diffusion region seems to be determined by the electrons trapped in the field‐reversal region, and the reconnection electric field is well explained by the nongyrotropic part of the pressure tensor of such electrons (Hesse et al., 1999).

## 5 Summary and Discussions

Using measurements from the four MMS spacecraft, the current sheet structure, including the characteristics of the electron velocity distribution function and the electric field, are studied during the EDR crossing event of 11 July 2017, 22:34:02 UT. Associated with the EDR crossing, electron distribution functions showed signatures of acceleration due to the out‐of‐plane electric field of the meandering electrons. The acceleration rate was consistent with the theoretical prediction by Bessho et al. (2014). The scale size of the current sheet of the inner EDR was consistent with the gyration scale size of electrons for the magnetic field at the edge of the diffusion region, as was predicted by theory (Kuznetsova et al., 1998; Hesse et al., 1999). Hasegawa et al. (2019) also obtained similar scale size of the EDR current sheet based on the reconstruction technique by adding electric field due to nongyrotropic pressure using the model by Hesse et al. (1999) to the two‐dimensional, time‐independent form of magnetohydrodynamic equation. These results add further quantitative supporting evidence that this EDR results from approximately 2‐D laminar reconnection (Genestreti et al., 2018; Nakamura et al., 2018; Torbert et al., 2018).

We showed that the observed electric field was comparable to the reconnection electric field from the nongyrotropic pressure term using the formula of Hesse et al. (1999). It should be noted, however, that for spacecraft not at the center of the current sheet, there should also be a contribution from the electron bulk flow inertia term. The convective term, *E*_M,IN_ = −(*m*_e_/*e*) *V*_eN_ (∂*V*_eM_/∂*N*), is not small within the EDR unless at the X‐line of 2‐D symmetric reconnection, where *V*_eL_ = *V*_eN_ = 0 and ∂ /∂*M = 0*. Using the time interval around the crossing of the X‐line in the *L* direction, 22:34:02.3–22:34:02.7 UT, we calculate the average values of the velocity and its gradient, <*V*_eN_> = 230 ± 220 km/s and <∂*V*_eM_/∂*N*> = −470 ± 149 km/s/km, and then estimate that the average inertia electric field is <*E*_M,IN_> = 0.6 ± 0.4 mV/m. Hence, it is still smaller than *E*_M,NG_ and should not be significant at the MMS locations. That the effect of *E*_M,IN_ relative to *E*_M,NG_ is still small as long as *E*_M_′ is comparable to *E*_M_, is obtained also in the PIC simulation profile by Nakamura et al. (2016). Yet it is interesting to note that the estimated electric fields, *E*_M,NG_, (solid and dashed‐dotted lines in Figures 4b–4d) tend to underestimate the electric field for MMS2 and MMS4, which are located farther away from the center of the current sheet, compared to MMS1 and MMS3. This difference is at least consistent with the effect of *E*_M,IN_ which becomes larger when off the equator.

The spatial dimensions of the inner EDR in antiparallel reconnection have been examined using PIC simulations, from which scaling laws based on the mass ratio were used to predict the results for a realistic mass ratio (Nakamura et al., 2016; Shay et al., 2007). Taking into account that the edge of the diffusion region is the point where the electric field corresponding to the Lorentz force becomes smaller than the reconnection electric field *E*_M_ (where *E*_M_′ becomes negative), Shay et al. (2007) found that the inner EDR scale size becomes smaller as the mass ratio becomes larger and predicted that for a mass ratio of 1,836 the inner EDR scale size, ∆_*L*_ ∼ 0.6 *d*_i_. This scale of inner EDR is indeed consistent with a PIC simulation with mass ratio of 1,836 performed by Goldman et al. (2011), judging from the profile of the reconnection electric field (Figure 3 from Goldman et al., 2011). Using the formula of Hesse et al. (1999) for obtaining λ_L_ discussed above, Nakamura et al. (2016) predicted λ_L_ ∼ 3*β*_e_^1/4^(*d*_i_ *d*_e_)^1/2^ based on a simulation with a different mass ratio. When the observed values: *β*_e_ = 0.1, *d*_i_ = 1,300 km, *d*_e_ = 31 km are used in their formula, however, we obtain a somewhat smaller extent of the diffusion region in the *L* direction, that is, ∆_*L*_ ∼ 0.1 *d*_i_ and λ_*L*_ ∼ 1.2 *β*_e_^1/4^(*d*_i_ *d*_e_)^1/2^. Although we cannot quantify the scale of the entire electron diffusion region further away from the X‐line in the same way as was done for the inner EDR determination, due to the limited time interval when we can follow the X‐line motion, the observed meandering electron signature in the Earthward outflow jet region discussed in section 3 and the extended region of thin‐current sheet support the results from simulations that EDR has a two‐scale structure (e.g., Shay et al., 2007).

The observed value of the vertical width of the inner electron diffusion region, *λ*_N,ave_ = 30 ± 7 km, on the other hand, was consistent with the prediction from the model of Shay et al. (2001), ∆_N_ ∼ *d*_e_ = 31 km, as well as with the model prediction from the simulation of Nakamura et al. (2016), λ_N_ ∼ 3*β*_e_^3/8^(1 + 0.15 *β*_e_^1/2^) *d*_e_ = 41 km. The good coincidence of the observed EDR size along *N* with the two different theoretical scaling laws based on electron inertia and electron gyration scales indicates also that *β*_e_ was about 1 at the edge of the EDR current sheet. This can be confirmed from the observed value at edge of EDR in the tailward flow region, as can be seen in the *β*_e_ dip close to 1 between the two vertical lines in Figure S1f.

Considering that the scale size of the EDR in the simulation is usually measured during steady state reconnection, the difference in the scale size along *L* between our observation and the simulations may suggest that the observed reconnection is still developing and that therefore the observed EDR has not reached the full scale. In fact Nakamura et al. (2018) found better agreement with the scale size of the observed EDR when such temporal evolution is taken into account. As shown in Figure 1, the crossing of the neutral sheet that took place ~2 s before the EDR crossing had no signature of a thin current sheet or electron jet, which we can interpret as due to the crossing of the outer edge of electron jet region near the X‐line. The rather stable electric field, *E*_M_, during this interval supports this interpretation of crossing a spatial structure rather than a temporal variation of the reconnection region. Extrapolating the spatial evolution of the X‐line shown in Figure 1j to ~2 s before EDR, it indicates that the electron outflow jet subsides within 400–600 km from the X‐line, that is, 0.3–0.5 *d*_i,_ from the X‐line. The entire EDR size, corresponding to the electron jet region, was shown to be about 0.6–1 *d*_i_ in PIC simulations with mass ratio 1,836 (Goldman et al., 2011; Le et al., 2016). The estimated scales of the electron jet region from observation are therefore also slightly smaller than those from the simulations.

It is interesting to note that this EDR event is preceded by another enhancement in the tailward flow and followed by a series of flux rope events (Stawarz et al., 2018; Teh et al., 2018; Torbert et al., 2018), as can be seen in Figure S1, suggesting a multiple reconnection event. The flux ropes showed different tilts in their orientation so that the current sheet goes beyond the 2‐D geometry further from the X‐line (Stawarz et al., 2018; Teh et al., 2018). This multiplicity of reconnection may be another reason for the smaller dimension of the inner EDR, making the effective gyroradius smaller due to the compressed normal magnetic field and may also be controlling the extent of the electron jet region. Nonetheless, the overall EDR signatures observed by MMS remarkably match the 2‐D reconnection picture expected from theory. The high‐time resolution measurements from four‐point observations enabled us for the first time to compare the reconnection parameters quantitatively with predictions from theory and simulation and to demonstrate how reconnection really works in space plasma.

## Supporting information



Supporting Information S1
