# Supplementary material for: Structure of the Current Sheet in the 11 July 2017 Electron Diffusion Region Event
Source: J Geophys Res Space Phys. 2019 Feb 27;124(2):1173–86. doi: 10.1029/2018JA026028 (PMC6472497; doi:10.1029/2018JA026028)
Supplement: Supplementary file 1 — Supporting Information S1 [file JGRA-124-1173-s001.pdf]

AGU PUBLICATIONS

Journal of Geophysical Research: Space Physics
Supporting Information for

Structure of the Current Sheet in the 11 July 2017 Electron Diffusion Region Event

Rumi Nakamura[1], Kevin J. Genestreti[1,2], Takuma Nakamura[1], Wolfgang Baumjohann[1], Ali Varsani[3], Tsugunobu Nagai[4], Naoki Bessho[5], James L. Burch[6], Richard E. Denton[7], Jonathan P. Eastwood[8], Robert E Ergun[9], Daniel J. Gershman[5], Barbara L. Giles[5], Hiroshi Hasegawa[4], Michael Hesse[10], Per-Arne Lindqvist[11], Christopher T. Russell[12], Julia E. Stawarz[8], Robert J. Strangeway[12], Roy B. Torbert[2,6]

[1] Space Research Institute, Austrian Academy of Sciences, Graz, Austria.
[2] Space Science Center, University New Hampshire, Durham, New Hampshire, USA.
[3] Mullard Space Science Laboratory, University College London, Dorking, United Kingdom.
[4] Institute of Space & Astronautical Science, Japan Aerospace Exploration Agency, Sagamihara, Kanagawa, Japan
.[5] NASA, Goddard Space Flight Center, Greenbelt, USA.
[6] Southwest Research Institute, San Antonio, Texas, USA. .
[7] Department of Physics and Astronomy, Dartmouth College, Hanover, New Hampshire, USA. .
[8] The Blackett Laboratory, Imperial College London, London, UK. .
[9] Department of Astrophysical and Planetary Sciences, University of Colorado, Boulder, Colorado, USA. .
[10] Department of Physics and Technology, University of Bergen, Norway. .
[11] Royal Institute of Technology, Stockholm, Sweden. .
[12] Department of Earth, Planetary, and Space Sciences, University of California, Los Angeles, USA. .

Contents of this file

Figures S1 to S4

Introduction

The supporting information contains event overview figure (Figure S1) from MMS3 and figures (Figures S2-S4) of electron velocity spectra near the X-line from the three MMS spacecraft (MMS 1, 2, and 4). Figures S2-S4 are produced in the same way as Figure 2 for MMS3 and have the same format as Figure 2 except for the different spacecraft.

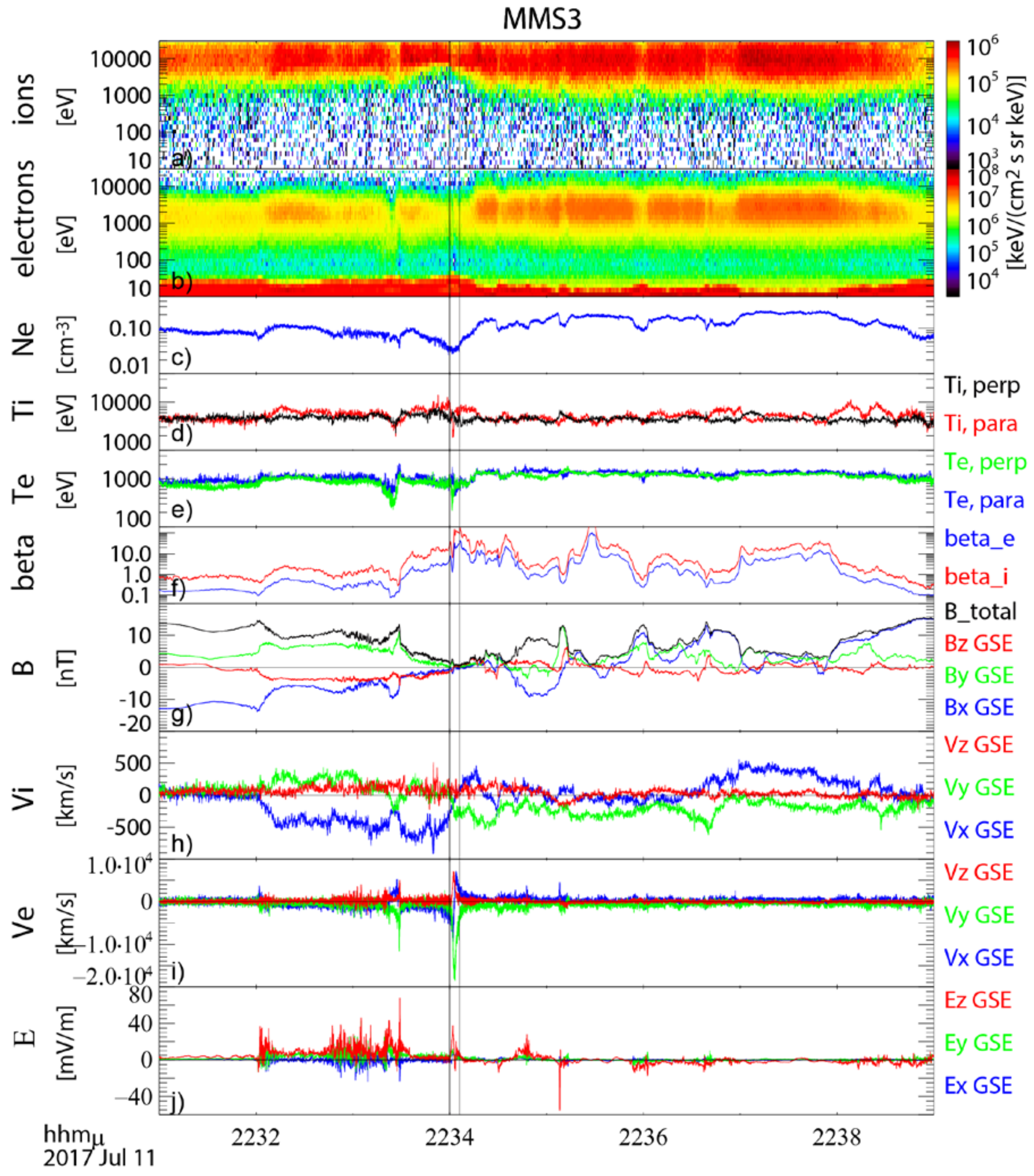

Figure S1. MMS3 observations during the flow reversal event. (a) ion and (b) electron energy spectra, (c) electron density, (d) perpendicular (black trace) and parallel (red trace) ion temperature, (e) perpendicular (green) and parallel (blue) temperature, (f) ion (red) and electron (blue) plasma beta, (g) magnetic field magnitude (black) and vector components, (h) ion and (i) electron velocity, (j) electric field. The *X*, *Y*, and *Z* components in the GSE coordinate system are plotted in (g-j) as blue, green and red traces, respectively. The EDR crossing studied in this paper took place between 22:34:00 and 22:34:06 as indicated by the vertical lines.

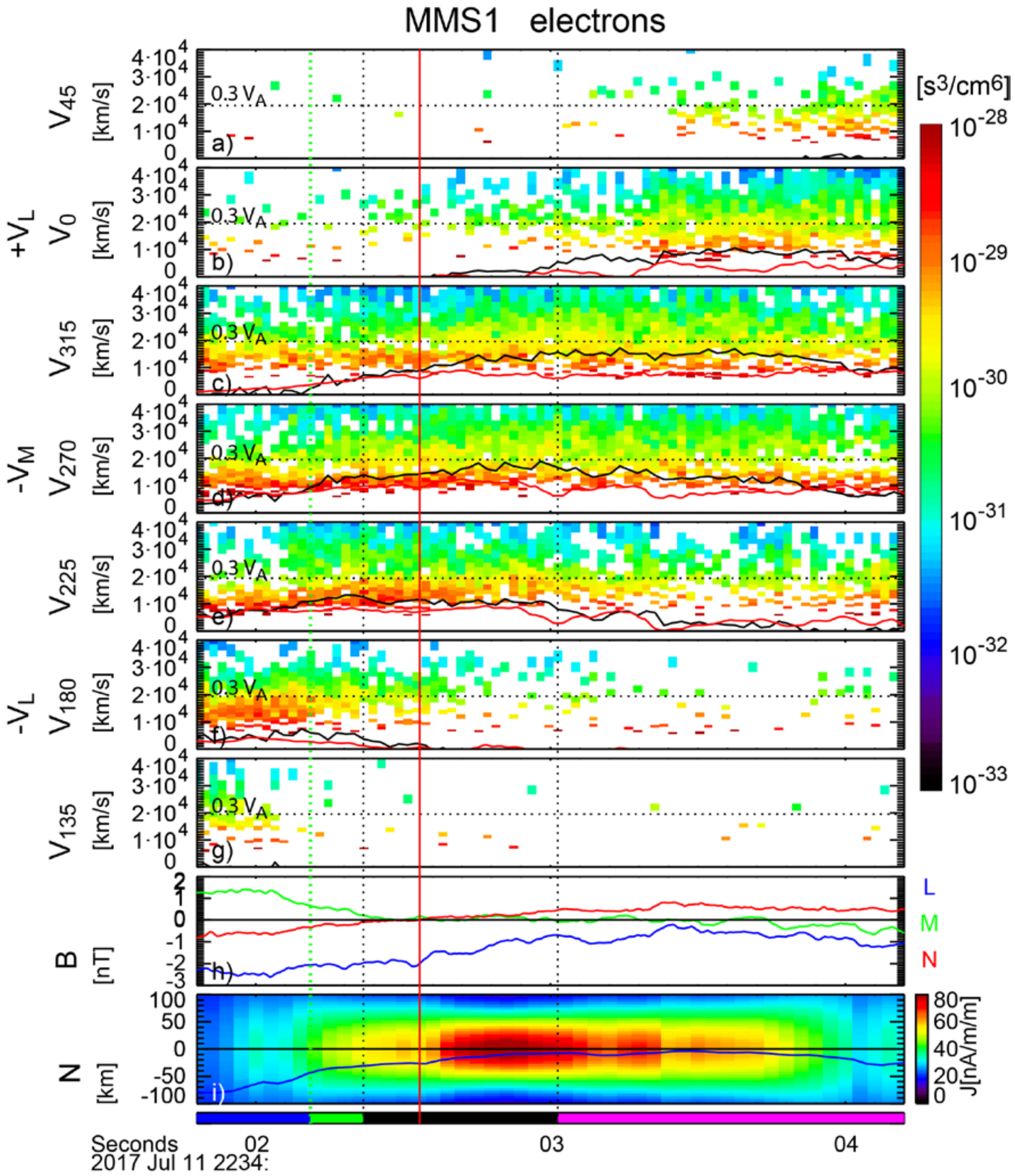

Figure S2. MMS1 electron velocity spectra near the X-line crossing and the location of the spacecraft relative to the current sheet. Velocity spectra cuts in directions within the *L-M* plane along the (a)V_{45}, (b)V_0 or +V_L, (c)V_{315}, (d)V_{270} or -V_M, (e)V_{225}, (f)V_{180} or –V_L, and (g)V_{135} directions shown in Figure 3a and explained in the text. (h) *L* (blue), *M* (green) and *N* (red) components of the magnetic field. (i) N coordinate of MMS3 within the model current sheet. In (a-g) the bulk velocity and the E×B drift components along the direction of the cuts are shown as black and red curves, and the horizontal dotted line indicates the velocity level of 0.3 V_A =19500 km/s. The vertical red line indicates the crossing of the X-line in the *L* direction by MMS 3 at 22:34:02.4 UT. The vertical dotted lines show the demarcation of the four regions with different characteristics of the velocity distribution function marked with bars at the bottom, representing

the region outside the thin current sheet (blue), the tailward outflow jet region (green), the inner EDR (black), and the Earthward outflow jet region (magenta)

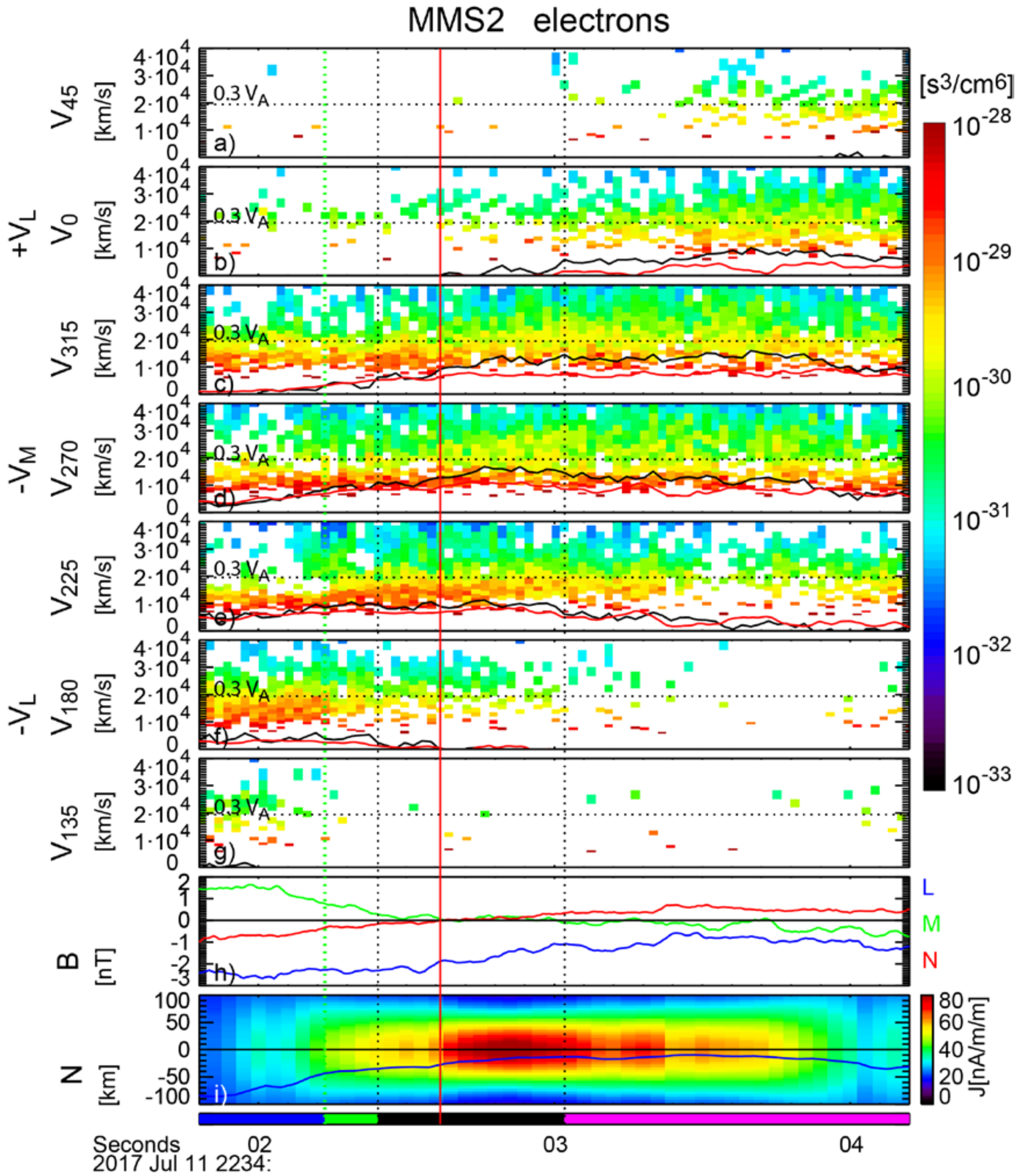

Figure S3. Same as Figure S2 except for MMS2.

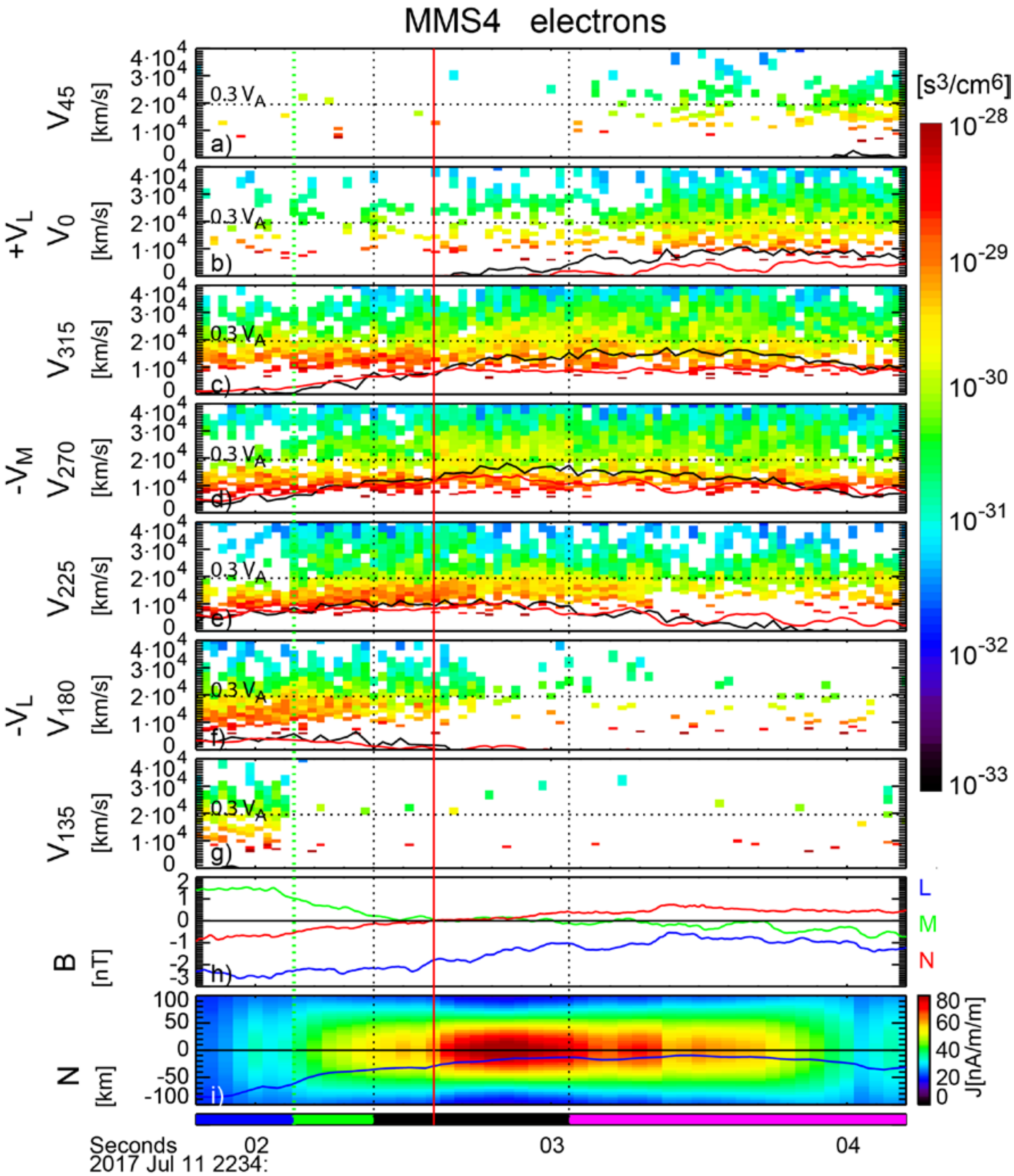

Figure S4. Same as Figure S2 except for MMS4.
